# A Novel Treatment Modality for Malignant Peripheral Nerve Sheath Tumor Using a Dual-Effect Liposome to Combine Photodynamic Therapy and Chemotherapy

**DOI:** 10.3390/pharmaceutics12040317

**Published:** 2020-04-02

**Authors:** Chin-Tin Chen, Po-Chun Peng, Tsuimin Tsai, Hsiung-Fei Chien, Ming-Jen Lee

**Affiliations:** 1 Department of Biochemical Science and Technology, National Taiwan University, No. 1, Sec. 4, Roosevelt Road, Taipei 10617, Taiwan; chintin@ntu.edu.tw (C.-T.C.); swaigod@hotmail.com (P.-C.P.); 2 Graduate Institute of Biomedical Materials and Engineering, Graduate School of Dentistry, Taipei Medical University, Taipei 11043, Taiwan; tmtsai00@gmail.com; 3 Division of Plastic Surgery, Department of Surgery, Taipei Medical University Hospital and College of Medicine, Taipei Medical University, Taipei 110, Taiwan; hfchien@gmail.com; 4 Division of Plastic Surgery, Department of Surgery, National Taiwan University Hospital and College of Medicine, National Taiwan University, Taipei 10012, Taiwan; 5 Department of Neurology, National Taiwan University Hospital, 7, Chung-Shan South Road, Taipei 10012, Taiwan; 6 Department of Neurology, National Taiwan University Hospital Yunlin Branch, 579, Sec. 2, Yunlin Road, Douliu City, Yunlin 640, Taiwan

**Keywords:** NF1, plexiform neurofibroma, MPNST, chemotherapy, liposome

## Abstract

Neurofibromatosis type 1 (NF1) is an inherited neurological disorder. Approximately 5–13% of NF1 patients may develop a malignant peripheral nerve sheath tumor (MPNST), which is a neurofibrosarcoma transformed from the plexiform neurofibroma or schwannoma. Given the large size and easy metastasis of MPNST, it remains difficult to be cured by either surgical or conventional chemotherapy. In this study, we investigated the possibility of combining photodynamic therapy (PDT) and chemotherapy to treat MPNST by using a dual-effect liposome (named as PL-cDDP-Ce6), in which a chemotherapeutic agent, cisplatin (cDDP), and photosensitizer, chlorine e6 (Ce6) were encapsulated in the same carrier. The cytotoxic effect of PL-cDDP-Ce6 against MPNST cells was significantly higher than cells treated with liposomal cDDP or Ce6 alone or in combination after light irradiation. Treatment with the dual-effect liposomes in mice bearing xenograft MPNST tumor reveals a significant increase in survival rate compared to those treated with liposomal cDDP and Ce6 in combination. Moreover, there is no weight loss or derangements of serum biochemistry. In conclusion, this study demonstrates the clinical potential and advantage of using this liposomal drug for the treatment of MPNST.

## 1. Introduction

Neurofibromatosis type 1 (NF1) is one of the autosomal dominant neurological disorders worldwide. Except for the skin pigmentation features, the hallmark of NF1 is the formation of benign nerve sheath tumors (neurofibromas) from schwann cells. Patients with cutaneous tumors appear during late childhood and develop progressively throughout life [1]. More complex neurofibromas such as plexiform neurofibroma surrounding the entire nerve affect as many as 30 percent of adults with NF1 who might normally appear during early childhood [2]. Total excision for these tumors is often challenging due to their size and location [3]. Moreover, they can undergo malignant transformation and have increased risk to become a malignant peripheral nerve sheath tumor (MPNST) [3]. Not only the plexiform neurofibroma, but also a few rare schwannomas can also be transformed into MPNST [4]. It has been reported that there is a 5–13% lifetime risk to develop MPNST for NF1 patients; however, the relative risk is a lot higher for those patients with large plexiform neurofibromas [3,5].

Presently, surgery is the principal therapeutic mode for plexiform neurofibromas. However, patients having large plexiform neurofibromas represent a surgical nodus. Meanwhile, there is a high recurrence rate if tumors are only partially resected due to the intense adherence or invasion into local tissue. Although chemotherapeutic agents were proved to be cytotoxic to the neurofibromas, systemic administration often causes severe side effects on the normal tissue. Ifosfamide and doxorubicin have been generally considered to be the first line chemotherapeutic agents for MPNST, which are unresectable and metastatic soft tissue sarcomas [6]. Nevertheless, even using the combination therapy of ifosfamide and doxorubicin, the response rate was only 21%, the median progression-free survival rate, 17 weeks and overall survival, 48 weeks, suggesting low response rate in conventional chemotherapy [7]. Up to date, the mainstream for managing these lesions is watchful waiting through long-term monitoring.

Cisplatin (cDDP) is a platinum-containing compound that acts as a DNA-crosslinking agent with further interfering with replication and transcription, leading to cell death [8]. Cisplatin alone or in combination therapy has been used to treat a variety of cancers, including the soft-tissue sarcoma. It has been shown that administration of high-dose doxorubicin and cisplatin could inhibit the cell growth of metastatic soft-tissue sarcomas and invasive thymoma [9]. In an NF1 derived recurrent MPNST, pretreatment of cisplatin combined with doxorubicin before the large resection, followed by fractionated radiotherapy was suggested to be a useful regimen [10]. Recently, cisplatin combined with doxorubicin and ifosfamide was also suggested as an effective adjunctive chemotherapy for the treatment of sporadic MPNST [11]. These findings indicate that cisplatin could be a candidate drug of choice to treat MPNST. However, its clinical use is limited due to the diverse side-effects in normal tissues such as neuro- and/or renal-toxicity. Liposomal cisplatin has been developed to reduce the side effects of free drug [12,13]. However, limited bioavailability in the tumor is a disadvantage of the liposomal cisplatin due to the slow kinetics of release and low drug-to-lipid molar ratios [12,14].

Photodynamic therapy (PDT) has been developed as a treatment modality, which combines photosensitizer (PS) and light with an appropriate wavelength to produce cytotoxic-free radicals. This therapeutic modality has been used in clinics to treat various medical conditions such as localized infections, skin diseases, premalignant and malignant disorders [15,16]. PDT has been employed in the management of nasopharyngeal carcinoma [17,18], sarcomas [19], and vascular anomalies [20,21]. PS activation by light irradiation generates reactive oxygen species, especially singlet oxygen, resulting in cellular damage and death [22]. Tumor ablation induced by PDT relates to the direct cell killing as well as damage to the exposed microvasculature. We have previously developed a PEGylated dual-effect liposome with doxorubicin or cisplatin encapsulated in the aqueous interior and chlorin e6 (Ce6) incorporated into the lipid bilayer [23,24]. Combining with optimal illumination scheme, the dual-effect liposome encapsulated with Ce6 and cisplatin (named as PL-cDDP-Ce6) could increase the tumor disposition of bioavailable cisplatin, resulting in significant therapeutic efficacy while reducing its toxicity [23,24]. In this study, we evaluate the feasibility and clinical potential of using PL-cDDP-Ce6 to treat MPNST both in vitro and in vivo. Meanwhile, we have previously shown that high level of serum soluble Axl (sAxl) was found in patients with plexiform neurofibroma and MPNST as compared to that of the patients with skin neurofibroma or controls, indicating the plasma level of sAxl could be a reliable biomarker in NF-1 management [25]. Thus, we also examined whether the level of sAxl correlates with the tumor reduction after PL-cDDP-Ce6 treatment.

## 2. Materials and Methods

### 2.1. Cell Lines

All MPNST cell lines, T265, ST8814, and S462-TY, derived from NF1 patients. These cell lines were a kind gift from Professor Nancy Ratner (Cincinnati Children’s Hospital, Cincinnati, OH, USA) and were grown as described [26,27]. The use of these three cell lines have previously been published and the detailed information could be found [26,27]. The cells were grown on the DMEM culture medium containing 10% FCS, 500 U/mL penicillin/streptomycin, 0.5 mM forskolin and 2.5 mg/dL insulin (Sigma-Aldrich, St Louis, MO, USA), 0.5 mM 3-ios-butyl-1-methylxanthine (Sigma-Aldrich, St Louis, MO, USA), and 10 nM b1-heregulin 177–244 (Mark Sliwkowski, Genentech Inc., San Francisco, USA). Cultured plates were coated with 1 mg/mL poly-L-lysine (Sigma-Aldrich, St Louis, MO, USA) and 4 mg/dL natural mouse laminin (Gibco). Cell cultures were maintained at 37 ℃ in a humidified atmosphere containing 10% CO_2_.

### 2.2. Preparation of PL-cDDP and PL-cDDP-Ce6

Liposomal PL-cDDP and PL-cDDP-Ce6 were prepared according to the method described previously [24]. Briefly, 1,2-Distearoyl-sn-glycero-3-phosphocholine, 1,2-distearoyl-sn-glycero-3-phosphoethanolamine-N-[methoxy(polyethylene glycol)]-2000 and cholesterol (10:0.2:5 molar ratio) were dissolved in ethanol. cDDP was dissolved in 0.9% (w/v) NaCl at 65 ℃. The ethanolic solution containing lipids was added into the cDDP mixture to prepare PL-cDDP. For preparing PL-cDDP-Ce6, Ce6 and lipids were dissolved in the ethanol before mixing with cDDP. The mixture was sonicated at 65 ℃ for 1 h, and then extruded through polycarbonate membranes with 100 nm pore size. Size exclusion chromatography was used to remove the untrapped lipids, cDDP and Ce6. Particle size distribution of liposomal drugs was analyzed using a particle sizer (SZ-100, HORIBA, Kyoto, Japan). The prepared PL-cDDP or PL-cDDP-Ce6 was suspended in 0.9% (w/v) NaCl and stored at 4 ℃ for further study as described before [24].

### 2.3. In Vitro Cytotoxicity of PL-cDDP-Ce6 against MPNST Cells

MTT assay was employed to examine the cytotoxicity of liposomal drugs on MPNST cells in vitro. Cells were cultured in the 96-well plates with 8 × 10^3^ cells in each well. Liposomal drugs were incubated for 2 h and then exposed to light irradiation for a fluence of 0.1 J/cm^2^. The light device is a home-made 662 nm diode laser with power intensity of 95 mW/cm^2^. After light irradiation, cells were incubated in complete medium at 37 ℃ for 24 h. For MTT assay, 0.4 mg/mL of MTT solution was added into the cells after removal of medium. The MTT-medium was eliminated after 2 h in dark. Viable cells convert the MTT into a purple colored formazan product with an absorbance wavelength at 570 nm. The cell viability was evaluated by the principle, MTT activity (%) = (mean absorbance of treated cells/mean absorbance of control cells) × 100%.

### 2.4. In Vivo Therapeutic Effect in Mice Bearing S462-TY Xenograft Tumor

Athymic nu/nu nude mice were purchased from BioLASCO Taiwan (Taipei, Taiwan). These nude mice were housed in the specific pathogen-free environment with food and water ad libitum. All the procedures and research protocols have been approved by the National Taiwan University Institutional Animal Care and Use Committee (IACUC). IACUC Approval code is 20180368 and the valid date is 1 August 2019. Mice aged 6–8 weeks, were subcutaneously injected with 3 × 10^6^ S462-TY cells diluted in 50% matrigel, into the left flank. Each study group contained five mice. A single dose of liposomal drugs diluted in 0.9% NaCl saline were intravenous injected into the mice when tumor size grew up to 100 mm^3^ or 1000 mm^3^. The control group was given 0.9% NaCl saline solution. After drug administration, light irradiation was applied onto the tumor at 2 and 12 h, respectively. The light source was a diode laser as described in Section 2.3. The irradiation time is around 17 minutes and 32 seconds for a fluence of 100 J/cm^2^.

A toxic effect was defined as the reduction of 20% of the original body weight. The tumor size and body weight were measured every three days. Tumor volumes were calculated using the relation V = (a^2^ × b)/2, with a being the smallest and b being the largest perpendicular diameters. The mouse was considered as death when the tumor volume grows over 2500 mm^3^. At the end of the study, mice were euthanized in accordance to the IACUC regulations.

### 2.5. Tissue Preparation for Histopathology, Complete Blood Cell Count, and Biochemical Analysis

Tumor-bearing mice treated with PL-cDDP-Ce6 were sacrificed 72 h after light irradiation. Blood samples collected through submaxillary cardiac puncture were used for complete blood cell count (CBC) and biochemical analysis. After perfusion, the mice, liver, heart, spleen, lung, and kidney were harvested. Tumor tissue was dissected and fixed in 10% formaldehyde for histopathological examination. The experiments for CBC, biochemistry, and histopathology were carried out by Taiwan Mouse Clinic, National Comprehensive Mouse Phenotyping and Drug Testing Center (Academia Sinica, Taipei, Taiwan).

### 2.6. ELISA of Soluble Axl Level

The measurement of sAxl was performed according to the method described previously [25]. 50–100 µL of whole blood from submandibular vein of the treated mice were collected at the time of analysis. The blood was stored in a tube coated with anticoagulant, EDTA. After spun with 3000 rpm for 15 minutes, the supernatant plasma was separated and then stored at −80 ℃ until use. The concentration of sAxl was detected using a commercial available kit from R&D System (catalogue No. DY154) and measured by a ELISA reader as described previously [25]. For each sample, three independent concentrations in duplicates were used to validate the assay accuracy of the measurement.

### 2.7. Statistics

Student’s t-test and two-way ANOVA were used to analyze the statistic differences in cell killing and tumor volume between groups, respectively. The survival rate was evaluated by Kaplan–Meier survival curve and the difference were analyzed by log-rank test. Statistical analysis was conducted using IBM SPSS Statistics software, version 17.0. The *p* values < 0.05 was considered statistically significant.

## 3. Results

### 3.1. The Anti-Tumor Efficacy of Free-cDDP or PL-cDDP in S462TY Xenograft Mouse Model

**Figure 1:**
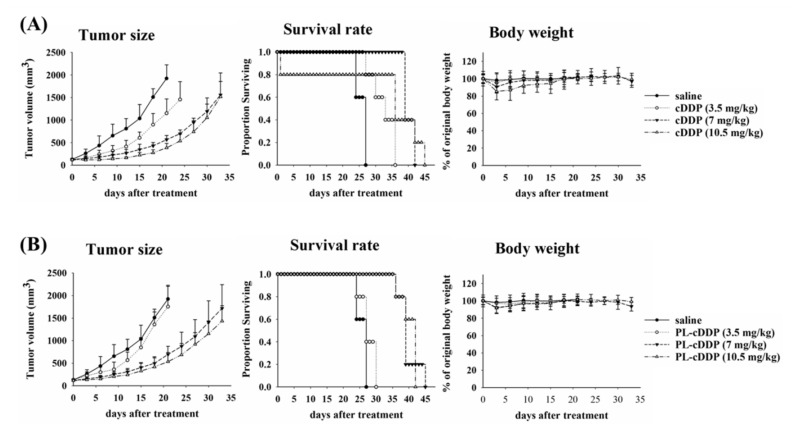
Therapeutic efficacy of free form (**A**) and liposomal (**B**) cisplatin (cDDP) on nude mice bearing human S462-TY malignant peripheral nerve sheath tumor (MPNST) tumor. The xenograft mice were subjected to variable doses of cDDP (3.5, 7 and 10.5 mg/kg) via intravenous injection. Left panel, tumor size; Middle panel, survival rate; Right panel, body weight. Data are presented as mean ± S.D. for each group. Four mice were used in the group received 10.5 mg/kg; while five mice were used in other groups.

To assess the possibility of using cisplatin (cDDP) to treat MPNST, variable dose of cisplatin was intravenously injected into the nu/nu nude mice bearing human S462-TY MPNST cells. These cells were created by passage of S462 MPNST cell line as xenografts [28]. Intravenous injection of cisplatin-free drug or PEGylated liposomal cDDP (PL-cDDP) was administrated into mice when the tumor grows to around 100 mm^3^. The tumor size of mice was monitored every 3 days after treatment. The mean size of the xenograft tumor treated with cDDP was significantly reduced in comparison with the untreated group (left panel of Figure 1A). Although treatment with high dose of cDDP demonstrated a high tumor reduction and proportion of survival, no significant difference was found between the two groups treated with 7 and 10.5 mg/kg cDDP, respectively (Figure 1A). The dose of cDDP to inhibit the tumor growth should be higher than 7 mg/kg for xenograft mice, which might be associated with a high frequency of side effect such as body weight loss (left panel of Figure 1A). Therefore, we further examined the therapeutic effect by using PL-cDDP. As shown in Figure 1B, similar therapeutic outcome was found in S462-TY tumor-bearing mice treated with PL-cDDP containing the corresponding dose of cDDP (Figure 1B).

### 3.2. The Cytotoxic Effect of PL-cDPP-Ce6 In Vitro against MPNST Cells

**Figure 2:**
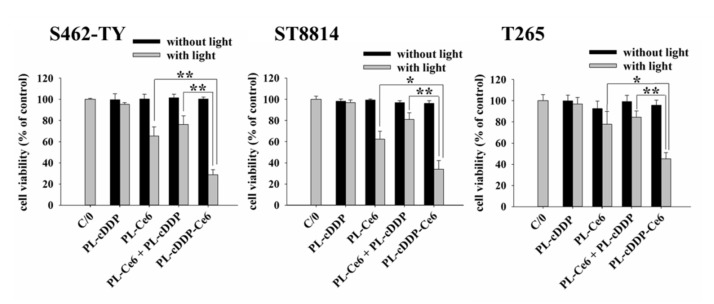
In vitro cytoxicity against S462-TY, ST8814, and T265 MPNST cells. Cells were incubated with different liposomal Ce6 (1 µg/mL), cDDP (2.8 µg/mL) or in combination for 2 h and then subjected to light irradiation (0.1 J/cm^2^). MTT assay was used to evaluate the cell viability 24 h after light irradiation (* *p* < 0.05, ** *p* < 0.01).

As shown above, a high dose of cDDP was required to exert anti-tumor effect in tumor-bearing mice treated with cDDP alone or PL-cDDP that systematic cytotoxicity remains a concern. Previously, we have shown that, combining with two doses of light irradiation, a single dose of PL-cDDP-Ce6 containing 3.72 mg/kg cisplatin and 1.75 mg/kg Ce6 results in significant tumor reduction in C26 tumor-bearing mice compared to the combination of PL-cDDP and PL-Ce6 [24]. To evaluate the possibility of using this liposome, PL-cDDP-Ce6 to treat MPNST, we first examined the cytotoxic effect of PL-cDDP-Ce6 on three MPNST cell lines, ST8814 and T265 and S462-TY cells. Figure S1 in the Supplementary Materials showed the characteristics of the PL-Ce6, PL-cDDP and PL-cDDP-Ce6 used in this study. As shown in Figure 2, no significant cytotoxicity was found in cells treated with PL-cDDP under the dose of 2.8 µg/mL. The viability of the three cell lines treated with PL-Ce6 alone or the combination of PL-cDDP and PL-Ce6 was about 60% to 80% after light irradiation. No significant difference was found between the cells treated with PL-Ce6 alone or PL-Ce6 and PL-cDDP in combination, indicating the cytotoxicity is from the Ce-6 mediated PDT. Meanwhile, the cellular viability was less than 40% in the group treated with PL-cDDP-Ce6 after light irradiation (Figure 2). The significant difference between the cells treated with PL-Ce6 alone and PL-cDDP-Ce6, suggesting an additive benefit of the therapeutic effect against MNPST cells by using PL-cDDP-Ce6.

### 3.3. The Antitumor Efficacy of PL-cDPP-Ce6 In Vivo against S462TY Xenograft Tumor

**Figure 3:**
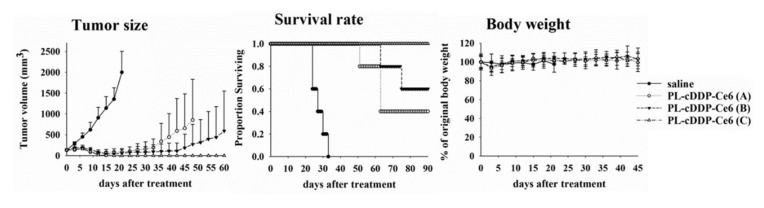
Therapeutic outcome of PL-cDDP-Ce6 in human S462-TY xenograft tumor-bearing mice. Mice were injected with one dose of PL-cDDP-Ce6 and received two dose of light irradiation (100 J/cm^2^) at 2 and 12 h post-drug administration, respectively. The dose of cDDP administrated in mice was 3.5 mg/kg and the dose of Ce6 varied under the doses of 1 (A), 1.25 (B) and 1.5 mg/kg (C). Left panel, tumor size; Middle panel, survival rate; Right panel, body weight. The presented data are mean ± S.D. for each group (N = 5).

To verify the therapeutic effect of PL-cDDP-Ce6 in vivo, we intravenously injected PL-cDDP-Ce6 containing different doses of Ce6 into the tail vein of the S462-TY xenograft mice with tumor size around 100 mm^3^. As performed previously, light irradiation (100 J/cm^2^) was applied onto the tumor at 2 and 12 h, respectively, after drug administration [24]. As shown in Figure 3, the tumor size increased by time in the saline control group. The mice were regarded as death when the size of tumor was greater than 2500 mm^3^. The longest survival of the mice in the control group was 33 days (left panel of Figure 3). However, compared to the control group administrated with saline, the levels of tumor regression in mice treated with the dual-effect liposome increased in proportional to the Ce6 amounts in PL-cDDP-Ce6 (3.5 mg/kg cDDP and various amount of Ce6). The Kaplan–Meier survival curve showed tumor-free in two and three out of five mice in the group treated with PL-cDDP-Ce6 containing 1 mg/kg and 1.25 mg/kg Ce6, respectively (middle panel of Figure 3). In fact, tumor-free was found in all mice treated with this dual-effect liposome containing 1.5 mg/kg Ce6 and lived longer than 90 days without tumor re-growth. As reported in the literature and the results in Figure 1A, body weight loss relates to the systemic side effect of cDDP. The body weight of mice did not change significantly in the PL-cDDP-Ce6 treated group during the treatment periods (right panel of Figure 3).

**Figure 4:**
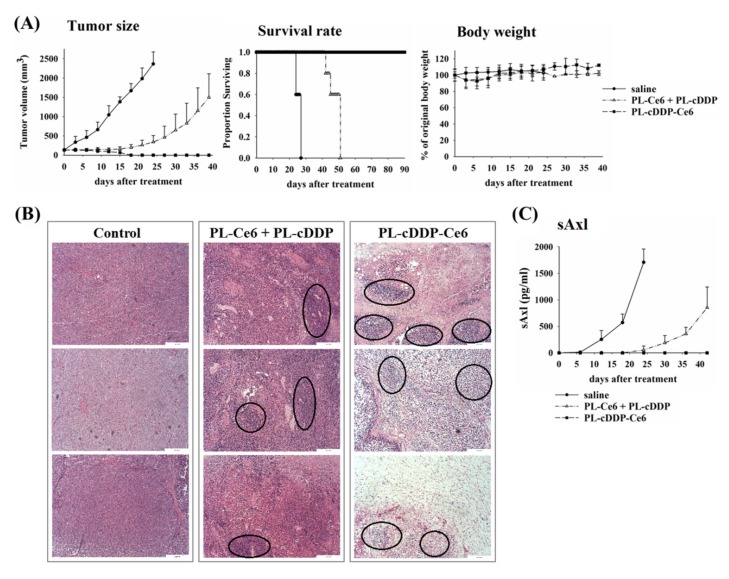
Therapeutic outcome and histological analysis of mice under the impact of PL-cDDP-Ce6 or PL-Ce6 and PL-cDDP in combination. Liposomal drugs (3.5 mg/kg of cDDP and 1. 5 mg/kg of Ce6) were intravenous injected into tumor-bearing mice. Then, light irradiation (100 J/cm^2^) was applied onto the S462-TY xenograft tumor at 2 and 12 h, respectively, post-drug administration. (**A**) After treatment, tumor size (left panel), survival curve (middle panel) and body weight (right panel) were recorded and analyzed. The data are expressed as mean ± S.D. for each group (N = 5). (**B**) S462-TY tumor-bearing mice were treated with saline (left panel), PL-Ce6 and PL-cDDP in combination (middle panel) and PL-cDDP-Ce6 (right panel). Tumor was harvested three days after treatment, then the tissue sections were stained with hematoxylin-eosin. The circles show the inflammatory necrotic tissues. (**C**) The levels of sAxl corresponding to the tumor size were analyzed against the days after treatment.

As shown in Figure 1, there is no significant therapeutic effect in mice treated with PL-cDDP (3.5 mg/kg). However, tumor-free was found in all mice treated with PL-cDDP-Ce6 (3.5 mg/kg of cDDP and 1.5 mg/kg of Ce6), indicating Ce6 mediated PDT might play an important role in this therapeutic approach. We, therefore, examined whether the combined use of PL-cDDP and PL-Ce6 can show similar therapeutic effect. As shown in Figure 4A, a significant reduction in tumor size and increased survival were found in mice treated with the combination of PL-cDDP and PL-Ce6. The tumor size of the xenograft mice receiving the combination of PL-cDDP and PL-Ce6 was comparative to the PL-cDDP-Ce6 treated group for two weeks after treatment (left panel of Figure 4A). However, tumor-free could only be found in the PL-cDDP-Ce6 treated mice but not the combination group. The survival curve demonstrated that all the mice received PL-cDDP-Ce6 was survived up to 90 days (middle panel of Figure 4A) and no significant body weight loss in these mice (right panel of Figure 4A). The representative histological tissue sections of the residual tumor revealed that more necrotic areas were found in mice treated with PL-cDDP-Ce6 (right panel of Figure 4B). However, only a small portion of necrotic areas could be found in mice treated with PL- Ce6 and PL-cDDP in combination (middle panel of Figure 4B). The AXL receptor tyrosine kinase has been implicated in the tumorigenesis of several cancers. Previously, we have shown that high level of the soluble fraction from the extracellular domain of AXL (sAXL) was found in patients with plexiform neurofibroma and with MPNST [25]. We therefore also examined the levels of sAxl in mice treated with liposomal drugs. As shown in Figure 4C, the tumor size of the treated mice was highly correlated with the plasma level of sAxl. In fact, the plasma sAxl was not found in the mice treated with PL-cDDP-Ce6 (Figure 4C).

Doxorubicin (Dox) has been used for treating MPNST, we therefore also evaluated whether the liposomal platform co-encapsulated with Dox and Ce6 (PL-Dox-Ce6) could be used to treat MPNST tumor. To evaluate the therapeutic efficacy of PL-Dox-Ce6, we prepared the PEGylated liposomes, PL-Dox-Ce6 and PL-Dox, which have the same phospholipid composition [23]. A single dose of PL-Dox-Ce6 or the combination of PL-Dox and PL-Ce6 was given to the nude mice bearing human S462-TY xenograft with tumor size greater than 100 mm^3^. After drug administration, light irradiation (100 J/cm^2^) were applied onto the tumor at 2 and 12 h, respectively. Complete tumor regression was found in three over six mice treated with PL-Dox-Ce6 but not in the group treated with PL-Ce6 and PL-Dox in combination (Figure S2, Supplementary Materials). These findings indicate that the anti-MPNST tumor effect of PDT and chemotherapy can be achieved using the dual-effect liposome. In fact, the concentrations for both photosensitizer and chemotherapeutic agents in dual-effect liposome are much lower than either PDT or conventional chemotherapy alone.

### 3.4. PL-cDDP-Ce6 Treatment for Larger MPNST Xenograft Tumor

**Figure 5:**
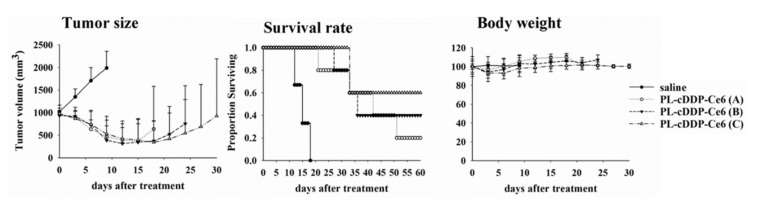
Therapeutical outcomes of PL-cDDP-Ce6 on nude mice bearing S462-TY xenograft tumor with average tumor size of 1000 mm^3^. A single dose of saline or PL-cDDP-Ce6 was injected into the tail vein of mice in each group (N = 5). The Ce6 dose administrated in mice was 1. 5 mg/kg and the cDDP dose varied, 1.43 mg/kg (A), 2.54 mg/kg (B), and 4.07 mg/kg (C). After drug injection, light irradiation (100 J/cm^2^) was applied onto the tumor at 2 h and 12 h, respectively. Left panel, tumor size; Middle panel, survival rate; Right panel, body weight. Data are presented as mean ± S.D. for each group.

The tumor size of plexiform neurofibroma is usually larger than that of cutaneous or subcutaneous benign neurofibroma. In addition to schwannomas, there are abundant extracellular matrix, blood supply, mast cells, and fibrous tissues in plexiform neurofibroma and MPNST. Therefore, we further investigated whether PL-cDDP-Ce6 has therapeutic efficacy to increase the survival rate of mice with larger size of tumor. To verfiy this argument, a single dose of PL-cDDP-Ce6 was intravenously injected into mice with S462-TY tumor size around 1000 mm^3^. Light irradiation (100 J/cm^2^) were applied onto the tumor at 2 and 12 h post-drug administration, respectively. Saline injection into the tumor-bearing mice was used as a control group. Compared to the rapid tumor growth in control group, tumor regression was significant in mice treated with PL-cDDP-Ce6 (Left panel of Figure 5). The overall survival rate significantly increased when the cDDP dose of PL-cDDP-Ce6 increased from 1.43 mg/kg to 4.07 mg/kg (middle panel of Figure 5). Tumor-free mice could be found in the mice treated with PL-cDDP-Ce6. In fact, the ratio of complete tumor regression was in proportion to the cDDP amounts encapsulated in the PL-cDDP-Ce6. There is no detective changes in the body weight of mice during the treatment, indicating no acute toxicity.

### 3.5. Safety Evaluation of PL-cDDP-Ce6

**Figure 6:**
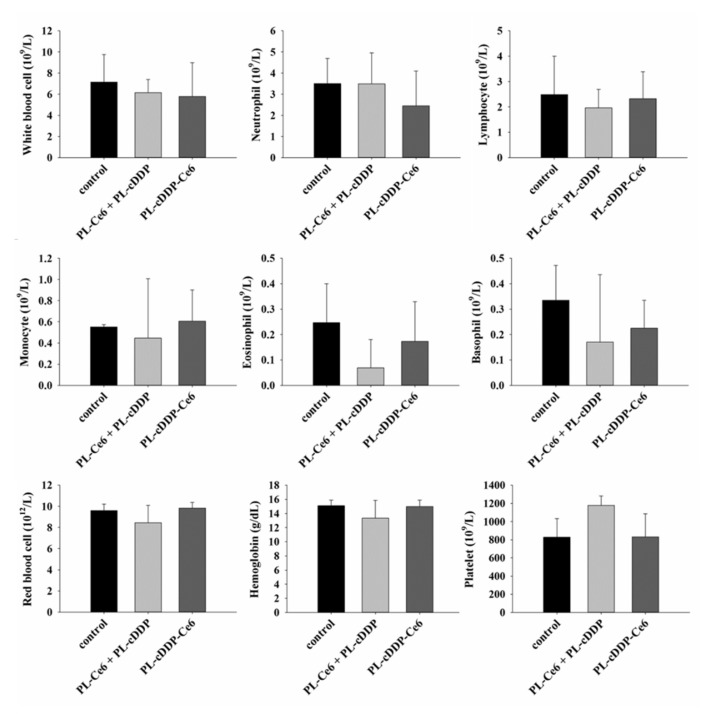
Hematology assay of white blood cells, neutrophil, lymphocytes, monocyte, eosinophil, basophil, red blood cells, hemoglobin and platelets 3 days post the treatment of PL-cDDP-Ce6 or PL-cDDP and PL-Ce6 in combination.

The internal organs (liver, heart, spleen, lung and kidneys) were removed to examine the in vivo safety of using PL-cDDP-Ce6. No weight loss or gain over 20% was found in the mice during the treatment. The activity and motor function of the mice treated with PL-cDDP-Ce6 were unremarkable. The gross inspection of the internal organs, heart, liver, spleen lung and kidneys was normal in both control and the PL-cDDP-Ce6 treated mice. There was no internal bleeding, organmegaly, significant inflammation, or fibrosis in the dissected tissues. To further examine the possible cytoxicity of PL-cDDP-Ce6, hematology and serum biochemical analysis were performed 3 days post the infusion of liposomal drug treatment. The counts of white blood cells (WBC) and other immune cells were recorded to investigate the immune response. We did not find a significant change in the WBC and other immune cell counts in the control and liposomal drugs treated groups (Figure 6). Hematotoxicity due to bone marrow suppression might be relevant to the administration of cDDP. Therefore, the count of red blood cell (RBC), hemoglobin (HB), and hematocrit (HCT) were recorded for analyzing the hematopoietic capacity. The data from the mice treated with either PL-cDDP-Ce6 or PL-Ce6 and PL-cDDP in combination were similar to that of saline control mice (Figure 6). Table 1 shows the function of liver and kidney after the treatment. The liver function was monitored by determing the serum level of aspartate aminotransferase (AST), lactate dehydrogenase (LDH) and alanine aminotransferase (ALT), whereas the levels of blood urea nitrogen (BUN) and creatinine (CRE) were used to evaluate the kidney function. It has been shown that liposomal drug preferentially accumulates in organs containing the macrophages of the reticuloendothelial system (RES), such as liver. Therefore, it could be expected to find a higher AST and LDH levels in animals treated with PL-cDDP-Ce6 or the combination of PL-cDDP and PL-Ce6 (Table 1). Although the levels of AST and LDH are somewhat higher in the treated mice than those of the controls, the comparison did not attain a significant level due to the larger range of standard deviation among the treated mice. In this regard, negligible changes in statistical significance were found in these serum factors between the saline-treated mice and liposomes-treated mice (Table 1). These findings provide solid evidence that the PL-cDDP-Ce6 infusion followed by light irradiation did not cause a derrangement of metabolism. This study demonstrates the combination of PDT and chemotherapy by using PL-cDDP-Ce6 could significantly enhance the anti-tumor therapeutic efficacy while reducing toxicity for the management of MPNST in clinical applications.

## 4. Discussion

In this study, we utilized a dual-effect liposome co-encapsulated with Ce6 and cDDP to treat MPNST, which is intractable for conventional treatments. Compared to PL-cDDP and PL-Ce6 alone or in combination, this PL-cDDP-Ce6 liposome synergistically increased the cytotoxicity against three MPNST cell lines (Figure 2). Meanwhile, its tumoricidal effect was also verified in S462-TY xenograft-bearing mice (Figure 3), and the tumor regression level correlated with the reduction of circulating biomarker, sAxl (Figure 4). Receptor tyrosine kinase activity is highly correlated with tumorigenesis. Our previous study suggests that the expression of the AXL receptor tyrosine kinase is significantly higher in plexiform neurofibroma and MPNST [25]. Further investigation identified that the extracellular portion of AXL (sAXL) can be found in serum/plasma from the patients, indicating the level of sAXL could be a surrogate biomarker for the tumor burden in NF1 patients. In this study, the tumor size (Figure 4A) was found to be highly correlated with the level of sAXL (Figure 4C) in both control and the xenograft mice treated with PL-cDDP-Ce6. These findings argue for the feasibility of using the sAxl level as a related marker for growth of plexiform neurofibroma or its derived malignancy, MPNST [25]. Furthermore, increased amounts of cDDP in the PL-cDDP-Ce6 liposome result in significant tumor regression in mice with original tumor size greater than 1000 mm^3^ (Figure 5). In fact, the ratio of tumor-free mice was proportional to the cDDP dose in this dual-effect liposome. Finally, we showed that there was no concomitant systemic toxicity using this PL-cDDP-Ce6 liposome (Figure 6 and Table 1). These results demonstrate the possibility of using this dual-effect liposome to treat MPNST with reducing the potential toxicity from chemotherapeutic agents.

In this study, a single dose of free or PL-cDDP (3.5, 7.0 and 10.5 mg/kg) for chemotherapy could temporarily suppress the tumor growth in xenograft MPNST mice (Figure 1). However, PL-cDDP-Ce6 mediated PDT and chemotherapy demonstrated its significant therapeutic efficacy. As shown in Figure 3, tumor-free was found in mice treated with PL-cDDP-Ce6 containing 3.5 mg/kg cDDP with various doses of Ce6. Although the therapeutic outcomes of PL-cDDP-Ce6 increased in proportion to the Ce6 amounts, the increased tumoricidal effect is unlikely related to the Ce6-mediated PDT only. This could be supported by the following two arguments. First, we have previously shown that no significant therapeutic effect could be found in PL-Ce6-mediated PDT for a 1.25 mg/kg dose of Ce6 [23]. Second, the combination of PL-Ce6 (1.5 mg/kg) and PL-cDDP (3.5 mg/kg) could only delay tumor growth but not to attain a tumor-free state (Figure 4). Previously, our study showed that a high concentration of liposomal drugs could be delivered to the tumor tissue due to the vascular damage induced by PDT. Furthermore, the sustain release of bioavailable cDDP from this dual-effect liposome could be deposited at the tumor region to kill tumor cells [24]. Therefore, this significant therapeutic efficacy most likely relates to the synergistic effect of PDT and chemotherapy, which could only be found by using dual-effect liposome rather than the combination of two liposomal drugs.

According to the data from Italian and German soft tissue sarcoma cooperative group, up to 40% of MPNST occur in the extremities, followed by trunk/retroperitoneal (38%) and head and neck region (21%) [29]. In this regard, light irradiation required for PDT at these tumor lesions are not problematic for MPNST treatment. Up to date, there is a clinical trial trying to employ PDT against benign dermal neurofibroma (ClinicalTrials.gov Identifier: NCT01682811; Sponsor: Harry T Whelan, MD). However, the result is still pending. Meanwhile, Hamdoon et al. used ultrasound-guided interstitial PDT to treat an unusual case with solitary neurofibroma on the neck without the clinical manifestations of NF1 [30]. The patient’s syndromes such as pain, dysphagia, and shortness of breath caused by neck mass was improved in post-PDT follow-up. The neck MRI revealed a significant reduction in the tumor size three months post the treatment. They concluded that current PDT provides an alternative option in cases where complete excision leads to an unjustifiably high risk of morbidity. Although PDT can effectively shrink the benign neurofibroma and control its progression, whether a similar effect on MPNST can be replicated is unknown. Meanwhile, a few problems still remain to be settled to employ PDT for the treatment of neurofibromas. The first is that the depth of light penetration is too low to eradicate deep-seated, large tumors such as plexiform neurofibroma. The second is the systemic administration of high-dose photosensitizer might cause damage to surrounding normal tissues after light irradiation. The dual-effect liposome used in the present study demonstrated a high tumoricidal effect due to the facilitated release of cisplatin against larger tumor after PDT (Figure 5). Since the breaking of the liposome only occurs in the illuminated area, the systemic side effects of chemotherapeutic agents can be mitigated. Meanwhile, the released cDDP from the dual-effect liposome could exert its tumoricidal effect against larger tumor, which could compensate for the drawback of PDT in treating MPNST.

In this study, no significant body weight loss could be observed in those mice administrated with dual-effect liposomes, indicating systemic side effects of cDDP are limited given the local release in the illuminated area. Grossly, the major organs remained unremarkable in those mice, which suggests that using dual-effect liposome to deliver the chemotherapeutic agents is a safe and reliable novel strategy to treat MPNST (Figure 6 and Table 1). The main concern in using cDDP is the possible platinum-induced peripheral neurotoxicity. It has been shown that the dose for intravenous chemotherapy of cDDP to treat human sarcoma or MPNST is 50–100 mg/m^2^ [10,31]. In this study, the overall survival of mice bearing a large size of MPNST tumor significantly increased when the drug dose of cDDP escalated from 1.43 mg/kg to 4.07 mg/kg, which is around 4.3 to 12.3 mg/m^2^ (Figure 5), suggesting low risk of neurotoxicity. In fact, there is no abnormal movement activities such as cycling, tremor or bradykinesia of the treated mice during the experiment. In this regard, administrating only one dose of PL-cDDP-Ce6 could be a safe and effective treatment modality for the clinical management of MPNST.

The present study has shown the potential of using dual-effect liposome for the management of MPNST. However, to bring this treatment modality as the first line of clinical treatment, protocols such as standardization of the dose of dual-effect liposomal drugs, illumination scheme, and number of treatment cycles need to be considered along with the lesion location, the thickness of the MPNST and the present or degree of tissue dysplasia. With its low risk of systemic side effects, convenience of use in outpatient settings, ability to treat large and recurrent lesions, and minimal invasiveness to the patients, dual-effect liposome has the potential to be one of the choices for managing plexiform neurofibroma or even MPNST in NF1 patients.

## 5. Conclusions

In this study, we demonstrated the therapeutic efficacy and safety of dual-effect liposome encapsulated with Ce6 and cDDP for treating MPNST tumors in vivo. Complete tumor eradication was found in larger tumor and the tumor-free ratio was in proportion to the cDDP amounts, suggesting the promising clinical potential of these liposomal drugs for managing MPNST.

## Figures and Tables

**Table 1:** Serum biochemical tests of aspartate aminotransferase (AST), alanine aminotransferase (ALT), creatinine kinase (CPK), lactate dehydrogenase (LDH), blood urea nitrogen (BUN), and creatinine (CRE). Data represented as mean ± SD of three mice per group.

	Control	PL-Ce6 + PL-cDDP	PL-cDDP-Ce6
**AST (U/l)**	86.7 ± 36.7	142.3 ± 93.9	173.7 ± 125.4
**ALT (U/l)**	39.7 ± 26.1	21.7 ± 4.0	40.3 ± 25.7
**CPK (U/l)**	144.3 ± 18.5	151.7 ± 59.5	161.3 ± 70.9
**LDH (U/l)**	1110.0 ± 211.7	3816.7 ± 3041.8	2933.3 ± 1325.1
**BUN (mg/dL)**	23.23 ± 2.89	22.43 ± 5.16	23.93 ± 2.15
**CRE (mg/dL)**	0.10 ± 0.00	0.37 ± 0.25	0.13 ± 0.06

## Supplementary Materials

The following are available online at https://www.mdpi.com/1999-4923/12/4/317/s1, Figure S1: Therapeutical efficacy of PL-Dox-Ce6 on nude mice bearing human S462-TY xenograft tumor.
