# Supplementary material for: A Novel Treatment Modality for Malignant Peripheral Nerve Sheath Tumor Using a Dual-Effect Liposome to Combine Photodynamic Therapy and Chemotherapy
Source: Pharmaceutics. 2020 Apr 2;12(4):317. doi: 10.3390/pharmaceutics12040317 (PMC7238030; doi:10.3390/pharmaceutics12040317)
Supplement: Supplementary file 1 [file pharmaceutics-12-00317-s001.pdf]

Supplementary Materials

A novel treatment modality for malignant peripheral nerve sheath tumor using a dual-effect liposome to combine photodynamic therapy and chemotherapy

Authors: Chin-Tin Chen, Po-Chun Peng, Tsuimin Tsai, Hsiung-Fei Chien and Ming-Jen Lee

Contact: mjlee@ntu.edu.tw

Fig. 1

	Particle size (nm)	cDDP encapsulation efficiency (%)	cDDP loading capacity (%)	Ce6 encapsulation efficiency (%)	Ce6 loading capacity (%)
**PL-cDDP**	120	11.0	1.6		

Fig. 2

	Particle size (nm)	cDDP encapsulation efficiency (%)	cDDP loading capacity (%)	Ce6 encapsulation efficiency (%)	Ce6 loading capacity (%)
**PL-cDDP**	120	11.0	1.6		
**PL-Ce6**	120			78.0	1.1
**PL-cDDP-Ce6**	120	10.9	1.6	78.7	0.6

Fig. 3

	Particle size (nm)	cDDP encapsulation efficiency (%)	cDDP loading capacity (%)	Ce6 encapsulation efficiency (%)	Ce6 loading capacity (%)
**PL-cDDP-Ce6 (A)**	120	10.9	1.6	77.5	0.5
**PL-cDDP-Ce6 (B)**	120	10.9	1.6	78.7	0.6
**PL-cDDP-Ce6 (C)**	120	11.6	1.8	78.2	0.8

Fig. 4

	Particle size (nm)	cDDP encapsulation efficiency (%)	cDDP loading capacity (%)	Ce6 encapsulation efficiency (%)	Ce6 loading capacity (%)
**PL-cDDP**	120	11.0	1.6		
**PL-Ce6**	120			78.0	1.1
**PL-cDDP-Ce6**	120	11.6	1.8	78.2	0.8

Fig. 5

	Particle size (nm)	cDDP encapsulation efficiency (%)	cDDP loading capacity (%)	Ce6 encapsulation efficiency (%)	Ce6 loading capacity (%)
**PL-cDDP-Ce6 (A)**	120	15.0	2.1	77.7	2.1
**PL-cDDP-Ce6 (B)**	120	12.9	1.8	77.3	1.1
**PL-cDDP-Ce6 (C)**	120	10.9	1.6	78.7	0.6

Fig. 6

	Particle size (nm)	cDDP encapsulation efficiency (%)	cDDP loading capacity (%)	Ce6 encapsulation efficiency (%)	Ce6 loading capacity (%)
**PL-cDDP**	120	11.0	1.6		
**PL-Ce6**	120			78.0	1.1
**PL-cDDP-Ce6**	120	11.6	1.8	78.2	0.8

Figure S1. The characteristics of PL-Ce6, PL-cDDP and PL-cDDP-Ce6 used in each figure of this study. Encapsulation efficiency: total entrapped drug/ total drug added; Loading capacity: total entrapped drug/ total liposome weight.

(A)

	Particle size (nm)	Dox encapsulation efficiency (%)	Dox loading capacity (%)	Ce6 encapsulation efficiency (%)	Ce6 loading capacity (%)
**PL-Dox**	150	66.5	12.6		
**PL-Ce6**	150			49.3	1.7
**PL-Dox-Ce6**	150	35.0	7.3	33.4	1.4

(B)

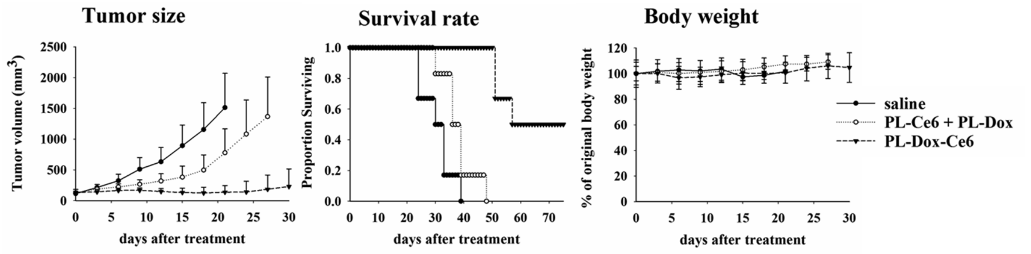

Figure S2. (**A**) The particle size, loading capacity and entrapment efficiency of PL-Ce6, PL-Dox and PL-Dox-Ce6. (**B**) The therapeutically efficacy of PL-Dox-Ce6 and the combination of PL-Ce6 and PL-Dox on nude mice bearing human S462-TY xenograft tumor. After liposomal drug injection, light irradiation was applied onto the tumor at 2 hr and 12 hr, respectively. The dose of Ce6 and Dox administrated in mice was 1.5 mg/kg and 7 mg/kg, respectively. Left panel, tumor size; Middle panel, survival rate; Right panel, body weight. Data presented are the mean ± S.D. for each group (N=6). Complete tumor regression was observed in 3/6 mice treated with PL-Dox-Ce6, resulting in apparent cure with clear superiority over the group treated with the combination of PL-Ce6 and PL-Dox.
